# Effects of a 12-Week Very-Low Carbohydrate High-Fat Diet on Maximal Aerobic Capacity, High-Intensity Intermittent Exercise, and Cardiac Autonomic Regulation: Non-randomized Parallel-Group Study

**DOI:** 10.3389/fphys.2019.00912

**Published:** 2019-07-17

**Authors:** Tomas Dostal, Daniel J. Plews, Peter Hofmann, Paul B. Laursen, Lukas Cipryan

**Affiliations:** ^1^Department of Human Movement Studies, Human Motion Diagnostic Centre, University of Ostrava, Ostrava, Czechia; ^2^Sport Performance Research Institute New Zealand (SPRINZ), Auckland University of Technology, Auckland, New Zealand; ^3^Exercise Physiology, Training and Training Therapy Research Group, Institute of Sports Science, University of Graz, Graz, Austria

**Keywords:** nutritional ketosis, graded exercise test, 30-15 intermittent fitness test, β-hydroxybutyrate, heart rate variability

## Abstract

**Purpose:**

The aim of this non-randomized parallel group study was to examine the 12 week effects of a very low-carbohydrate high-fat diet (VLCHF) on maximal cardiorespiratory capacity, high-intensity interval training (HIIT) performance, and cardiac autonomic regulation.

**Methods:**

Twenty-four recreationally trained participants allocated to either a VLCHF (*N* = 12) or a habitual diet (HD; *N* = 12) group completed 12 weeks of a diet and exercise (VLCHF) or an exercise only intervention (HD). Maximal graded exercise tests (GXT) were performed at baseline, after 4, 8, and 12 weeks. A supervised HIIT session and the 30-15 Intermittent Fitness Test (30-15_IFT_) were conducted once a week.

**Results:**

Total time to exhaustion (TTE) in both GXT and 30-15_IFT_ *largely* increased in both VLCHF (*p* = 0.005, BF_10_ = 11.30 and *p* = 0.001, BF_10_ ≥ 100, respectively) and HD (*p* = 0.018, BF_10_ = 3.87 and *p* = 0.001, BF_10_ ≥ 100, respectively) groups after 12 weeks. Absolute maximal oxygen uptake ($\dot{V}$O_2max_) was not changed in both groups but relative $\dot{V}$O_2max_ increased in VLCHF in concert with reductions in body mass (66.7 ± 10.2–63.1 ± 8.5 kg). Cardiac autonomic regulation did not reveal any between-group differences after 12 weeks. VLCHF diet induced an increase in β-hydroxybutyrate, which tended to normalize during the intervention period.

**Conclusion:**

The 12 week VLCHF diet did not impair high-intensity continuous or intermittent exercise lasting up to 25 min, nor did it impair maximal cardiorespiratory performance or autonomic nervous system (ANS) activity.

## Introduction

The very-low carbohydrate high fat (VLCHF) diet is a nutritional approach consisting of restricted carbohydrate (CHO) consumption and increased fat intake, with protein consumption and total energy intake preserved (Feinman et al., 2015). These changes contrast a traditional Western high CHO diet and cause a shift toward using more fat as a fuel source (Paoli et al., 2015), with promising potential shown for treating chronic metabolic and cardiovascular diseases (Mckenzie et al., 2017; Miller et al., 2018).

The VLCHF diet approach may also be of interest for conditioning coaches seeking methods of enhancing sports performance. The positive effects of the VLCHF diet on aerobic performance in sufficiently VLCHF diet adapted athletes have been described (Zajac et al., 2014; Paoli et al., 2015; Volek et al., 2015), however, studies still suggest that CHO may be the more critical substrate needed for high-intensity exercise performance (Burke, 2015; Burke et al., 2017). For example, Urbain et al. (2017) showed a reduction in absolute $\dot{V}$O_2peak_ and peak power (by 2.4 and 4.1%, respectively) during a maximal incremental cycling test following a 6 week VLCHF diet intervention. However, this study lacked a control group for comparison. Thus, consensus has not been reached regarding the longer-term impact of the VLCHF diet on high-intensity exercise performance.

Many especially believe that a VLCHF diet has a detrimental effect on performance during exercise in the very high intensity domain (i.e., above critical power). For example, Lima-Silva et al. (2013) showed in six healthy, physically active men that a short-term VLCHF diet (48 h) significantly (*p* < 0.05) reduced total time to exhaustion (TTE; 3.0 ± 0.2 min vs. 3.7 ± 0.3 following low-CHO vs. habitual diets, respectively) during all-out supramaximal cycling exercise (115% maximal oxygen consumption). In moderately trained men, unlike a habitual moderate- to high-CHO diet, a 6 week moderate protein, high fat diet (30% protein, 61% fat) was associated with a significant decrease in mean and peak power during the first bout of a Wingate test (538–426 W and 879–786 W, respectively) (Fleming and Sharman, 2003). In contrast, 400-m sprint performance, $\dot{V}$O_2peak_ and one-repetition maximum back squat were not adversely influenced after adoption of a 12 week ketogenic diet in recreationally trained CrossFit trainees when compared to a control group (Kephart et al., 2018). As well, Cipryan et al. (2018) showed after a 4 week VLCHF diet that individuals were able to perform high-intensity interval training (HIIT) with similar cardiorespiratory and RPE responses as a control diet group and without any indication of a performance compromise.

High-intensity interval training is an effective training method for enhancing cardiorespiratory, metabolic, and neuromuscular performance (Buchheit and Laursen, 2013; Tschakert and Hofmann, 2013) and can be efficiently used for managing and controlling a number of common metabolic diseases (Cassidy et al., 2017; Gillen and Gibala, 2018). HIIT includes several bouts of high-intensity exercise above critical speed/power, interspersed with recovery periods, and allows athletes to maintain long periods of time above 90% of $\dot{V}$O_2max_ (Buchheit and Laursen, 2013). Substantial dependence on anaerobic glycolytic energy contribution is required by default for HIIT. HIIT performance potential can be assessed using the 30-15 Intermittent Fitness Test (30-15_IFT_), which involves a graded, intermittent, shuttle field test (involving changes of direction), resulting in near maximal running velocity, with substantial energy provided by anaerobic sources during the last stages of the test (Buchheit, 2008; Buchheit and Laursen, 2014). However, the effect of longer-term carbohydrate restriction on HIIT performance and resulting physiological responses are not well documented.

The influence of dietary changes or physical interventions on physiological adaptation can be indirectly assessed through monitoring of the cardiac autonomic nervous system (ANS) activity via resting heart rate variability (HRV) measurement (Young and Benton, 2018). This non-invasive method enables assessment of the cardiac ANS status (Plews et al., 2016). To the best our knowledge, however, the effect of VLCHF diet adaptation on cardiac ANS activity has not been studied.

As mentioned, CHO availability is believed to be necessary to enable exercise performance in the high-intensity domain (e.g., >critical power) (van Loon et al., 2001). At first glance, we might assume that lowered CHO intake leads to lower CHO availability and consequent impairment of anaerobic metabolism and associated performance (Langfort et al., 1997; Yeo et al., 2011; Lima-Silva et al., 2013). In light of limited research examining prolonged adaptation periods to VLCHF diets (Cipryan et al., 2018), the purpose of the present study then was to examine the effects of a 12 week VLCHF diet intervention on HIIT performance and cardiac ANS activity, as monitored via morning resting HRV and the 30-15_IFT_.

## Materials and Methods

### Participants

**FIGURE 1:**
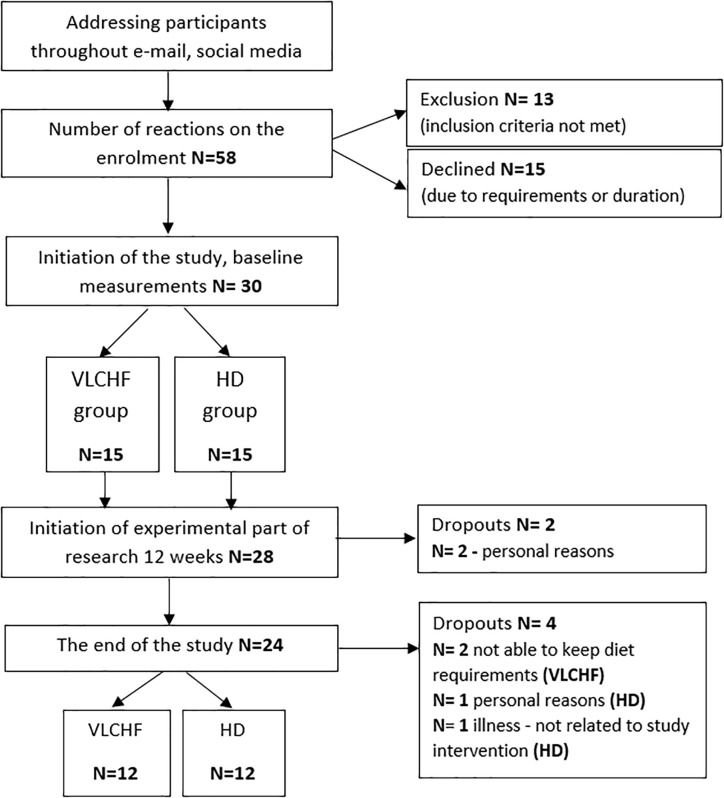
Study flow chart. VLCHF/HD, very low-carbohydrate high-fat diet/habitual diet.

In total, 30 healthy and moderately trained individuals were recruited through leaflet distribution in the local city area and via social media (Figure 1). Participants needed to be aged between 18 and 35 years, non-smokers, performing regular exercise at the recreational level (defined as having no specific sport training specialty and engaging in less than 60 min of moderate intensity exercise three times per week), and without experience to the low-carbohydrate high-fat diet. Exclusion criteria included having any overt cardiovascular, metabolic, respiratory or other chronic disease, acute or chronic musculoskeletal impairment prohibiting exercise training or testing, any medication or diet supplement, or any specific dietary restrictions (e.g., vegan) for at least 6 months prior. Participants were divided into either a very low-carbohydrate high-fat diet group (VLCHF; *N* = 15) or habitual diet group (HD; *N* = 15) according to subject preference. Alcoholic beverages and dietary supplements were restricted for the intervention period.

### Experimental Design

**FIGURE 2:**
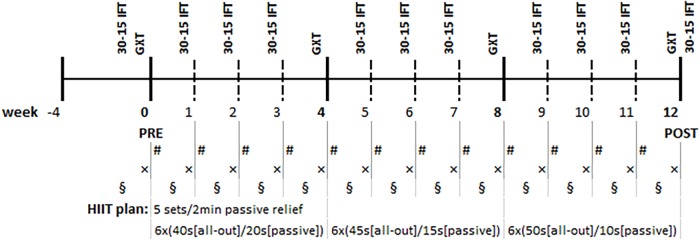
Research timeline. GXT, Graded Exercise Test; 30-15 IFT, 30-15 Intermittent Fitness Test (every Thursday/Friday); #, supervised High-Intensity Interval Training (HIIT) session (every Monday/Tuesday), ×, capillary blood draw for the glucose and β-hydroxybutyrate analysis (every Thursday/Friday), §, heart rate variability measurement (at least three times per week).

A non-randomized, two-group, parallel design was used. Twenty-four participants allocated to either a VLCHF or a HD group completed 12 weeks of diet and exercise (VLCHF) or exercise only intervention (HD). The detailed participants’ characteristics are presented in Table 1. Participants in the HD group were instructed to maintain their habitual diet (which was controlled by food diaries). Four participants (two from each group) were not able to meet the study requirements and did not complete the study (Figure 1). Diet, exercise and HRV were monitored 1 month before the intervention to obtain baseline levels. The laboratory graded exercise test (GXT) was performed at baseline, after 4, 8, and 12 weeks. A supervised HIIT session and 30-15 Intermittent Fitness Test (30-15_IFT_), separated by at least 48 h, were conducted each week (Figure 2).

**Table 1:** Characteristics of the participants.

	VLCHF (*N* = 12)		HD (*N* = 12)	
		
Male:Female	3:9		4:8	
Age (years)	25.3 ± 2.0		23.9 ± 3.8	
	**PRE**	**12 weeks**	**PRE**	**12 weeks**
	
Body mass (kg)	66.7 ± 9.8	63.1 ± 8.1	72.7 ± 15.0	71.8 ± 14.7
Skeletal muscle mass (kg)	29.2 ± 4.8	28.9 ± 4.5	30.9 ± 6.3	30.6 ± 6.3
Fat mass (kg)	14.4 ± 4.8	11.5 ± 4.1	17.5 ± 8.9	17.1 ± 8.4
Fat mass (%)	21.5 ± 4.9	18.3 ± 5.4	23.4 ± 8.4	23.2 ± 7.9
Fat mass on trunk (kg)	7.2 ± 2.7	5.5 ± 2.4	8.7 ± 4.7	8.5 ± 4.4
Total body water (kg)	38.1 ± 5.7	37.6 ± 5.4	40.3 ± 7.5	39.9 ± 7.5


### Diet Intervention

Participants in the VLCHF group were instructed to restrict carbohydrate consumption to a maximum 50 g/day (Feinman et al., 2015). Protein and fat were permitted to be consumed *ad libitum*. However, participants were encouraged to focus on increasing fat consumption in order to compensate for the reduced energy caused by carbohydrate restriction. Participants received detailed guidance concerning the VLCHF diet and its practical application from an experienced dietitian prior to the trial. Participants were provided with a sample diet and unlimited access to diet consultancy at any time during the experimental period. Participants from both groups were instructed to track their self-reported diet daily using a dietary analysis application^1^. A capillary blood sample was drawn from a finger for the measurement of β-hydroxybutyrate (FreeStyleOptium Neo, Oxon, United Kingdom) once a week to confirm compliance with the dietary regimen.

### Exercise Intervention

Participants were instructed to perform 3–5 training sessions per week. Two of these included a supervised HIIT session and the 30-15_IFT_. The remaining required training sessions (1–3 per week) were home-based and of an endurance format (e.g., running, cycling, sport games). All training sessions were recorded by heart rate monitor (Polar M400, Kempele, Finland).

The supervised HIIT sessions lasted approximately 40 min in total and consisted of a 4 min warm-up followed by 5 × 6 min sets separated by 2 min recovery. Each training set was composed of three short interval exercises and three strength/core exercises. Each 1 min bout was formed by progressively increasing the work/rest ratio as follows: weeks 1–4, work/rest ratio = 40 s/20 s; weeks 5–8, 45 s/15 s; weeks 9–12, 50 s/10 s (Figure 2).

### Graded Exercise Test

Prior to the start of the intervention, and then again in 1 month intervals (four times in total), participants underwent a laboratory GXT on a motorized treadmill (Rodby RL 2000E) in order to determine maximum aerobic power ($\dot{V}$O_2max_), the second ventilatory threshold (VT_2_), total time to exhaustion (TTE_GXT_), and respiratory exchange ratio (RER). The GXT protocol started at 7.0 km/h and running speed was increased by 1.5 km/h every 4 min (inclination remaining at 1%) conducted to volitional exhaustion. Expired air was continuously monitored to analyze O_2_ and CO_2_ concentrations during the GXT by the use of a breath-by-breath system (Blue Cherry, Geratherm Medical AG, Germany). $\dot{V}$O_2max_ was determined as the highest average O_2_ consumption measured over a 30 s period. Gas-exchange measurements were also used to quantify the second ventilatory threshold (VT_2_). VT_2_ was defined as a sharp increase in ventilation (VE) accompanied with an increase in both VE/VO_2_ and VE/VCO_2_. HR was measured using a chest belt monitor (Polar Electro H9, Kempele, Finland). Peak lactate was analyzed from capillary blood obtained from a finger immediately after GXT cessation. Participants also expressed their perceived effort on the 20-point Borg scale. All sessions were conducted in the morning, at least 3 h after their last meal and in a thermally controlled laboratory (21°C, 40% relative humidity). Each participant performed their laboratory sessions during similar morning hours (±30 min), and were advised not to participate in vigorous activity 24 h prior. Body mass and composition were determined using a bioelectrical impedance analyser (InBody770, Seoul, South Korea) before the GXT.

### 30-15 Intermittent Fitness Test

The 30-15 Intermittent Fitness Test (30-15_IFT_) was performed once a week (at least 6 days apart) in indoor conditions (Buchheit, 2008). The test consists of 30 s shuttle runs interspersed by 15 s passive recovery periods. Run speed began at 8 km/h for the first 30 s run and was increased by 0.5 km/h every 45 s stage thereafter. Heart rate was monitored throughout the test. Participants evaluated their perceived exertion using the same 20-point Borg scale immediately after exercise cessation.

### Heart Rate Variability

Participants performed a 60 s HRV measurement in a daily manner upon waking up whilst breathing spontaneously in a supine position (Plews et al., 2017). Participants were obliged to remain resting in a supine position at least 2 min before the HRV measurements. A Bluetooth chest belt (Polar H7 Bluetooth, Kempele, Finland) paired with a smartphone application Elite HRV (Ashville, NC, United States) were used for daily HRV measurement. The raw beat-to-beat (RR) data were acquired from Elite HRV and further processed for counting rMSSD variable as follows: RR intervals were corrected for artifacts according to two criteria. First, consecutive RR intervals were removed when they differed by more than 75% from the previous one. Additionally, outliers were removed by including only RR intervals that were within less than 25% of the first quartile and within more than 25% of the third quartile. This technique avoids over-correcting, a problem of the widely employed removal of consecutive RR intervals differing by more than 25% for individuals with very high beat-to-beat variability (Plews et al., 2017). The calculated rMSSD data (Camm et al., 1996) were log-transformed (ln) as a non-uniformity of error was expected (Plews et al., 2012). A 7 days ln rMSSD average was used for the cardiac autonomic regulation assessment, where at least three HRV measurements were recorded (Plews et al., 2016). The participants began to measure HRV 1 month before the intervention. However, the baseline rMSSD level was based on the last ten HRV measurements taken immediately prior to the intervention.

### Statistical Analysis

Descriptive statistics are displayed as mean ± SD. All parameters were tested for normal distribution using the Shapiro–Wilk test. Data were assessed for practical significance using standardized differences in the mean (Cohen’s *d*) which were calculated using the pooled standard deviation. Threshold values for the Cohen’s *d* were <0.20 (*trivial*), 0.20–0.49 (*small*), 0.50–0.79 (*medium*), ≥0.80 (*large*) (Cohen, 1988). 95% Confidence Limits were expressed for Cohen’s *d*. The two-tailed paired Student’s *t*-test (or Mann–Whitney test when significant deviation from normality) was used for the within-group time comparisons. The two-tailed independent Student’s *t*-test (or Wilcoxon signed-rank test when significant deviation from normality) was used for the between-group comparisons. In all cases, *p* < 0.05 were taken as the level of statistical significance.

Bayes factor (BF_10_) were calculated for differences between groups as well as for the before vs. during intervention comparisons using Cauchy prior (location 0, scale 0.707). Visual prior vs. posterior check and Bayes factor robustness check were considered. Bayes factor quantify relative evidence for the null and alternative hypothesis. Evidence categories for the BF_10_ were <1/100 *decisive evidence* for H_0_, 1/100-1/30 *very strong evidence* for H_0_, 1/30-1/10 *strong evidence* for H_0_, 1/10-1/3 *moderate evidence* for H_0_, 1/3-1 *anecdotal evidence* for H_0_, 1 *no evidence*, 1–3 *anecdotal evidence* for H_1_, 3–10 *moderate evidence* for H_1_, 10–30 *strong evidence* for H_1_, 30–100 *very strong evidence* for H_1_, >100 *decisive evidence* for H_1_ (Jeffreys, 1961). Statistical analyses were performed using JASP (version 0.9.1.0).

## Results

The between-group differences of the diet regime are shown in Table 2. The total energy and macronutrients intake were similar in both groups before the intervention. The macronutrient intake did not change in the HD group during the intervention period. Although the total energy significantly decreased (ES ± 95% CI: 0.69 ± 0.63, *p* = 0.035) in the HD group there were no between-group differences in total energy intake during the 12 week intervention period. According to the study aim, participants in the VLCHF group substantially reduced their CHO intake and increased their lipid intake (both decisive evidence for the large change). Changes in the body weight and body composition in both groups are shown in Table 1. Although energy intake slightly increased in the VLCHF group, body weight decreased by 6.4% and remained constant in the HD group. Body composition significantly changed only in the VLCHF diet group. Fat mass and trunk fat mass significantly decreased by 20.2 and 23.7%, respectively, after 12 weeks of diet in the VLCHF group only (Table 1).

**Table 2:** Diet and training characteristics.

			HD vs. VLCHF diff.			HD vs. VLCHF diff.		BEFORE vs. DURING diff.	
						
	Group	Before Mean ± SD	Cohen’s *d* (95% CI)	*p*-value^a^	During Mean ± SD	Cohen’s *d* (95% CI)	*p*-value^a^	Cohen’s *d* (95% CI)	*p*-value^b^
CHO (g)	VLCHF HD	194 ± 43 202 ± 42	0.18 (-0.64, 1.00)	0.661	40 ± 6 201 ± 49	4.59 (2.55, 6.60)^∗∗∗∗^	<0.001^c^	3.83 (2.07, 5.59)^∗∗∗∗^ 0.02 (-0.54, 0.59)^#^	<0.001 0.938
Proteins (g)	VLCHF HD	79 ± 21 85 ± 34	0.02 (-0.44, 0.46)	0.976^c^	113 ± 24 82 ± 30	-1.15 (-2.01, -0.27)^∗^	0.010	-1.22 (-2.00, -0.41)^∗∗^ 0.39 (-0.20, 0.97)	0.002 0.201


Lipids (g)	VLCHF HD	71 ± 17 70 ± 23	-0.14 (-0.55, 0.33)	0.608^c^	149 ± 26 67 ± 16	-3.87 (-5.25, -2.47)^∗∗∗∗^	<0.001	-3.17 (-4.65, -1.67)^∗∗∗∗^ 0.21 (-0.37, 0.77)	<0.001 0.492


Energy (kJ)	VLCHF HD	7523 ± 1541 7817 ± 1583	0.19 (-0.63, 1.00)	0.656	8206 ± 1322 7458 ± 1726	-0.49 (-1.29, 0.33)	0.246	-0.43 (-1.04, 0.20) 0.69 (0.05, 1.31)	0.185 0.035


TTT (min)	VLCHF HD	211 ± 131 219 ± 131	-0.06 (-0.86, 0.74)	0.877	174 ± 76 170 ± 57	0.05 (-0.75, 0.85)	0.896	0.33 (-0.29, 0.76) 0.41 (-0.19, 1.00)	0.339^c^ 0.179


HR_peak_ (% HR_max_)	VLCHF HD	92 ± 7 89 ± 6	0.46 (-0.36, 1.26)	0.276	95 ± 4 95 ± 4	-0.06 (-0.86, 0.74)	0.884	-0.76 (-1.39, -0.10) -1.66 (-2.53, -0.76)^∗∗∗∗^	0.024<0.001


HR_avg_ (% HR_max_)	VLCHF HD	73 ± 7 72 ± 7	0.06 (-0.75, 0.86)	0.893	78 ± 5 78 ± 6	-0.07 (-0.87, 0.73)	0.870	-1.26 (-2.01, -0.47)^∗∗∗^ -1.98 (-2.96, -0.97)^∗∗∗∗^	0.001<0.001


*Before, 4 weeks period before the intervention; During, 12 weeks intervention period; CI, confidence interval; VLCHF, very low-carbohydrate high-fat diet group; HD, habitual diet group; CHO, carbohydrate. TTT, average total training time per week; HR_*peak*_, peak heart rate; HR_*avg*_, average heart rate. Cohen’s *d* – the effect size for the between-group or Before vs. During difference. For the *t*-test, effect size is given by Cohen’s *d*, for the Wilcoxon test, effect size is given by the matched rank biserial correlation. ^*a*^Two-tailed *t*-test/Mann–Whitney Rank Sum Test, ^*b*^two-tailed paired *t*-test/Mann–Whitney Rank Sum Test, ^*c*^Mann–Whitney Rank Sum Test performed due to normality test (Shapiro–Wilk) or Equal Variance Test (Brown–Forsyth) failed at *p* < 0.05. The likelihood of the effect is shown (^∗^moderate, ^∗∗^strong, ^∗∗∗^very strong, ^∗∗∗∗^extreme evidence favors the alternative hypothesis; ^#^moderate evidence favors the null hypothesis).*

**FIGURE 3:**
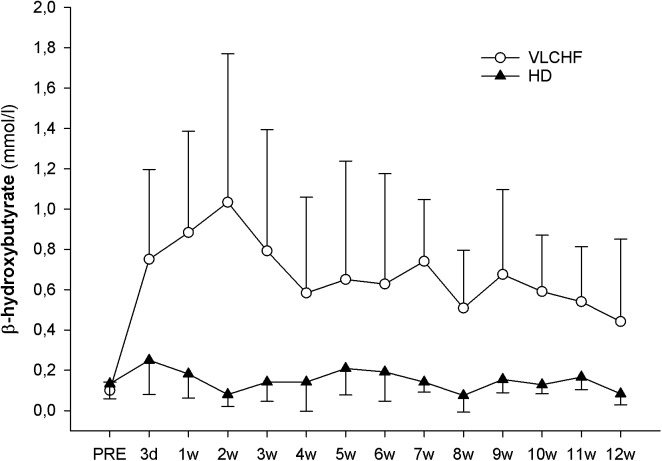
Capillary β-hydroxybutyrate concentrations.

The concentration of β-hydroxybutyrate (βHB) increased substantially in the VLCHF group within the first days of the diet intervention. The βHB concentration achieved its highest peak at the end of the 2 weeks (1.1 ± 0.7 mmol/l) but tended to normalize toward the end of the 12 week intervention period (Figure 3).

Total number of HIIT sessions (including 30-15_IFT_) through the experimental part of the research was 22 sessions. The smallest number of training sessions across all participants was 17 (*N* = 1). Average attendance was 20.4 ± 1.4 (92.8 ± 6.5%) training sessions for the VLCHF group and 20.1 ± 1.2 (91.3 ± 1.3%) training sessions for the HD group. The usual reasons for missed training sessions were lack of time or other personal reasons. The volume and exercise intensity were similar in the between-group comparison before as well as during the intervention period. However, there was very strong to decisive evidence for a large increase of the exercise intensity during the intervention period for both the VLCHF and the HD group (Table 2).

### Graded Exercise Test

**FIGURE 4:**
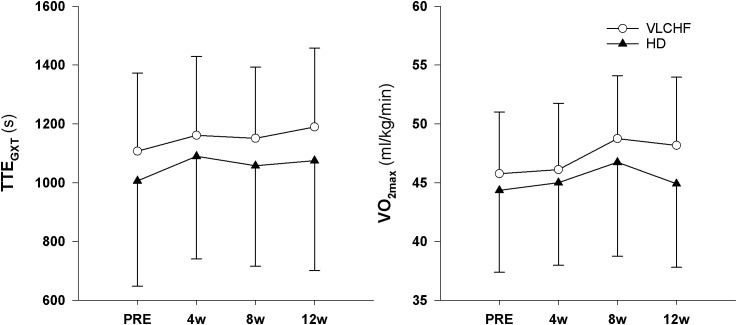
Total time to exhaustion (TTE_GXT_) and maximal oxygen consumption ($\dot{V}$O_2max_) in the GXT.

We found *very strong evidence* (BF_10_ = 11.30) for a *large* Total Time to Exhaustion (TTE_GXT_) increase in the VLCHF group (ES ± 95 CI: 1.03 ± 0.70, *p* = 0.005) and *moderate evidence* (BF_10_ = 3.87) for a *large* TTE_GXT_ increase in the HD group (ES ± 95 CI: 0.86 ± 0.70, *p* = 0.018) after 12 weeks. $\dot{V}$O_2max_ (ml/kg/min) also increased *largely* (ES ± 95 CI: 0.91 ± 0.67, *p* = 0.009, BF_10_ = 6.25) in the VLCHF group despite a *moderate evidence* for no significant (*p* = 0.591) change in the HD group. However, there was *moderate evidence* (BF_10_ = 0.29 and 0.32 for the VLCHF and HD group, respectively) for no changes in both groups when $\dot{V}$O_2max_ was expressed in absolute values (l/min). RER at maximal running speed decreased *largely* (ES ± 95 CI: -1.11 ± 0.76, *p* = 0.004, BF_10_ = 12.29) in the VLCHF group whereas it did not change in the HD group (ES ± 95 CI: -0.06 ± 0.57, *p* = 0.835, BF_10_ = 0.29) at the end of the intervention. The complete GXT results are shown in Figure 4 and Table 3. Average group values of lactate in the rest state decreased by 40% from 1.2 ± 0.3 (mmol/l) at the start of the intervention to 0.8 ± 0.3 (mmol/l) in VLCHF and no difference in HD at the end of the 12 weeks intervention. Average group values of lactate after the GXT decreased by 4.5% from 9.3 ± 2.2 (mmol/l) at the start of the intervention to 8.9 ± 2.4 (mmol/l) in VLCHF and by 16% from 9.6 ± 3.0 (mmol/l) to 8.1 ± 3.1 (mmol/l) at the end of the 12 weeks intervention.

**Table 3:** Graded exercise test results.

	VLCHF (*N* = 12) mean ± SD	PRE vs. week 12			HD (*N* = 12) mean ± SD	PRE vs. week 12			diff. in % change after 12 weeks		
					
		Cohen’s *d* (95% CL)	*p*-value^a^	BF_10_		Cohen’s *d* (95% CL)	*p*-value^a^	BF_10_	Cohen’s *d* (95% CL)	*p*-value^b^	BF_10_
**TTE_GXT_ (s)**		1.03 (0.30, 1.72)	0.005	11.30		0.86 (0.14, 1.54)	0.018	3.87	0.27 (-0.56, 1.09)	0.530	0.44
PRE	1108 ± 266				1006 ± 358						
Week 12	1190 ± 268				1075 ± 374						
**VO_2max_ (ml/kg/min)**		0.91 (0.22, 1.57)	0.009	6.25		0.16 (-0.41, 0.73)	0.591	0.32	-0.53 (-1.34, 0.29)	0.207	0.68
PRE	45.8 ± 5.2				44.4 ± 6.9						
Week 12	48.2 ± 5.8				44.9 ± 7.1						
**VO_2max_ (l/min)**		-0.02 (-0.59, 0.54)	0.940	0.29		0.13 (-0.44, 0.70)	0.657	0.32	0.15 (-0.65, 0.95)	0.718	0.39
PRE	3.05 ± 0.57				3.16 ± 0.78						
Week 12	3.04 ± 0.58				3.20 ± 0.87						
**HR_max_ (bpm)**		0.07 (-0.52, 0.66)	0.817	0.31		-1.06 (-1.76, -0.33)	0.004	13.27	-1.27 (-2.16, -0.36)	0.006	70.41
PRE	192.5 ± 8.1				190.5 ± 6.9						
Week 12	193.1 ± 7.0				185.5 ± 7.0						
**VT_2_ (% VO_2max_)**		-0.51 (-1.13, 0.13)	0.121	0.89		0.02 (-0.54, 0.59)	0.936	0.29	0.43 (-0.40, 1.25)	0.314	0.56
PRE	95.6 ± 2.3				93.9 ± 3.2						
Week 12	94.4 ± 1.7				94.3 ± 1.9						
**RER**		-1.11 (-1.86, -0.33)	0.004	12.29		-0.06 (-0.63, 0.51)	0.835	0.29	1.07 (0.18, 1.93)	0.019	3.30
PRE	1.03 ± 0.05				1.01 ± 0.08						
Week 12	0.95 ± 0.05				1.01 ± 0.06						
**RPE**		0.54 (-0.08, 1.14)	0.089	1.07		0.69 (0.21, 0.90)^c^	0.003^c^	>100	0.97 (0.11, 1.81)	0.026	2.56
PRE	17.8 ± 1.5				16.1 ± 1.7						
Week 12	18.8 ± 1.2				18.7 ± 1.0						


*VLCHF, very low carbohydrate high fat diet roup; HD, habitual diet group; TTE_*GXT*_, total time to exhaustion; VO_*2max*_, maximal oxygen consumption; VT_*2*_, second ventilatory threshold; RER, respiratory exchange ratio; RPE, Borg’s rating of perceived exertion. ^*a*^Two-tailed paired *t*-test, ^*b*^two-tailed independent *t*-test, ^*c*^Mann–Whitney test due to significant (*p* = 0.05) deviation from normality, effect size is given by the rank biserial correlation.*

### 30-15 Intermittent Fitness Test (30-15_IFT_)

**FIGURE 5:**
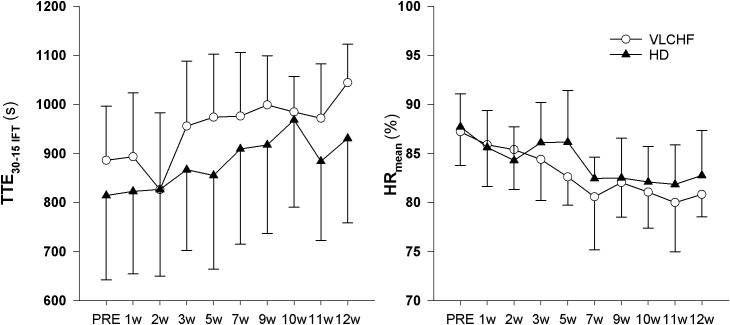
Total time to exhaustion (TTE_30-15IFT_) and mean heart rate (HR_mean_) during the 30-15_IFT_.

We found *decisive evidence* that TTE_IFT_ *largely* increased in both VLCHF and HD groups after 12 weeks (ES ± 95% CI: 1.93 ± 0.97 and 1.50 ± 0.84, respectively, *p* = 0.001 and BF_10_ ≥ 100 for both groups). Simultaneously with the TTE_IFT_ increase, HR_mean_ *largely* decreased in both VLCHF and HD groups after 12 weeks (ES ± 95 CI: -1.86 ± 1.00 and -1.26 ± 0.77, *p* = 0.001 and 0.001, BF_10_ ≥ 100 and 35.42, respectively) (Figure 5). There was *decisive evidence* for an RPE increase in both VLCHF (Mean ± SD: PRE 16.83 ± 1.85, 12 w 19.42 ± 0.79, ES ± 95% CI: 1.00 ± 0.00, *p* = 0.002, BF_10_ ≥ 100) (*note for VLCHF group: p*-value is from Wilcoxon signed-rank; effect size is given by the matched rank biserial correlation due to deviation from normality) and HD (Mean ± SD: PRE 16.67 ± 1.30, 12 w 19.08 ± 0.67, ES ± 95% CI: 1.61 ± 0.89, *p* = 0.001, BF_10_ ≥ 100) groups.

### Heart Rate Variability

**FIGURE 6:**
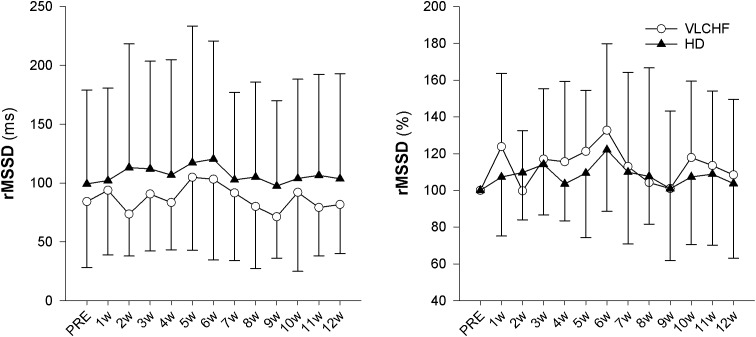
The time course of rMSSD during the intervention period, expressed in absolute units (ms) or as change (%) from the PRE score.

The vagal related HRV parameter rMSSD did not change during the monitored period. There is *moderate evidence* for a trivial effect in both study groups (ES ± 95 CI: -0.06 ± 0.60 and -0.16 ± 0.59, *p* = 0.856 and 0.611, BF_10_ = 0.30 and 0.33, respectively) (Figure 6).

## Discussion

The purpose of this controlled non-randomized study was to evaluate the effects of a 12 week non-energy-restricted VLCHF diet in healthy young individuals on maximal cardiorespiratory and HIIT performance as well as cardiac autonomic regulation. The adequate compliance to our diet intervention is highlighted by increased resting capillary blood βHB concentrations (Figure 3) and reduced RER during the GXT (Table 3). We found that the 12 week CHO intake restriction and fat-rich diet did not adversely impact on high-intensity continuous or intermittent performance. Interestingly, ßHB tended to normalize toward the end of the 12 week intervention period, potentially reflecting enhanced cellular uptake subsequent to adaptation. Additionally, as far as we know, this is the first study to report on resting cardiac autonomic response to the VLCHF diet intervention across 12 weeks. We showed that this predictor of training status (Buchheit, 2014) remained unaffected by the VLCHF diet intervention. Another interesting effect was the decrease in body mass, and specifically trunk fat mass, in the VLCHF diet group, despite unchanged energy intake (slight increase).

### The VLCHF-Induced Metabolic Shift

The 12 weeks exposure to the VLCHF diet in the present investigation is considered sufficient for inducing nutritional ketosis and also achieving an adequate adaptation to the VLCHF diet, which typically takes about 3–4 weeks (Volek et al., 2015). The rapid increase in capillary blood βHB concentration during the first 2 weeks (from a baseline value of ≤0.1–1.03 ± 0.74 mmol/l) and the maintenance of the elevated βHB level in the range of 0.5–3.0 mmol/L (Volek and Phinney, 2012) throughout the entire VLCHF diet intervention, provides evidence of established nutritional ketosis (Figure 3). These βHB concentration changes and subsequent dynamics correspond with previously reported studies (Mckenzie et al., 2017; McSwiney et al., 2017). The gradual decrease in βHB concentration shown after the 2 weeks of the VLCHF diet intervention, which tended to normalize, may be the result of enhanced mitochondrial function and capacity for ketone body utilization (Miller et al., 2018). Another possible explanation for the tendency for normalizing βHB concentration toward baseline conditions is an increase in gluconeogenetic glucose production (Veldhorst et al., 2009) supported by an increased protein intake during the intervention period (Table 2). Additionally, some suggest that this increase in gluconeogenesis leads to higher energy expenditure due to a diet-induced thermogenic increase of 20–30% (Keller, 2011). In contrast, this effect was absent from the study of Webster et al. (2016) in trained cyclists performing a 6 month high-fat diet. Nevertheless, such a thermogenesis effect is suggested to explain the weight loss shown in the VLCHF diet group even despite their higher energy intake compared to the pre-intervention period. The *strong evidence* for the *large* decrease of maximal RER during GXT at maximal running speed in the VLCHF group (Table 3) also indicates increasing whole-body rates of fat oxidation and accompanying reductions in CHO utilization. Previous studies examining VLCHF diets have reported similar findings for the RER response (Burke et al., 2017; Urbain et al., 2017). The increased utilization of fat and concomitant decrease in CHO oxidation during exercise can be associated with a number of different mechanisms, including the ability of skeletal muscle to transport, store, and oxidize free fatty acids, or alternatively, suppression of pyruvate dehydrogenase activity and glycogenolysis (Yeo et al., 2011).

### Performance Outcomes

**FIGURE 7:**
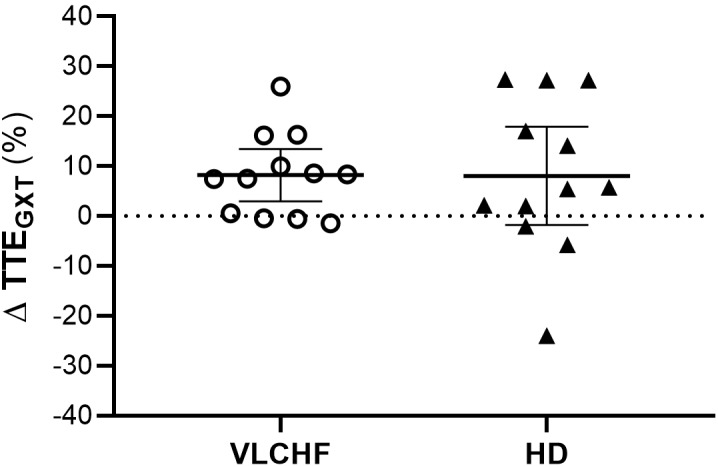
Individual responses of total time to exhaustion in GXT (TTE_GXT_).

**FIGURE 8:**
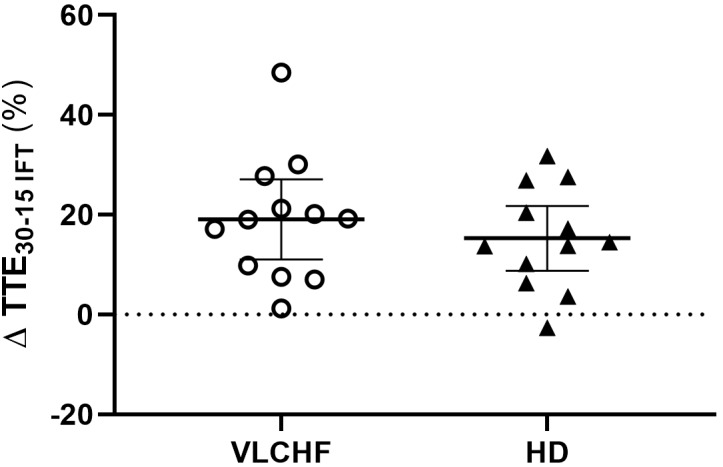
Individual responses of total time to exhaustion in 30-15_IFT_ (TTE_30-15IFT_). The means and 95% CI are shown. The outlier at this was not included in the group statistics (Table 3).

Total time to exhaustion in both GXT and 30-15_IFT_ *largely* increased in both VLCHF and HD group after the 12 weeks period. Additionally, our data showed no between-group differences in the TTE increase percentage changes between PRE vs. 12 week time points across the VLCHF and HD group for GXT (*p* = 0.530, BF_10_ = 0.44; Table 3) and 30-15_IFT_ (*p* = 0.428, BF_10_ = 0.47). These results are in line with Heatherly et al. (2018) who showed no significant difference in a 5 km time trial performance between their low- and high-carbohydrate diet groups. Further, average values of peak GXT lactate in VLCHF at the end of the 12 week intervention were similar to average peak values at the beginning; a finding which is in line with the study by Burke et al. (2017). A possible explanation may be that individuals can adapt to this type of diet by synthesizing glucose from others substrates (Volek et al., 2016). Similar to the study of McSwiney et al. (2017), we found no negative effects of the VLCHF diet on exercise during the 12 week intervention period, which is in contrast to the results of Burke et al. (2017), who indicate a negative impact on 10 km race time in elite walkers after 3 weeks of a VLCHF compared to a HD. Our results are also in conflict with general sports nutrition recommendations that emphasize the necessity for dietary CHO intake and sufficient availability to enable high-intensity exercise performance (Burke and Hawley, 2018). The glycogen content of the working muscle has been considered for several decades as a determinant for the capacity to perform high-intensity exercise (Bergström et al., 1967). When fat oxidation is intentionally increased through a shift of the macronutrient intake, CHO substrate pools are reduced. It has been shown that exercise economy (increased oxygen demand for a given speed) might also be diminished when conforming to a VLCHF diet (Burke et al., 2017). Of note, the findings of impaired exercise performance have usually been presented after only short periods on the VLCHF diet, which unlikely allows participants fully to adapt sufficiently to the dietary CHO reduction and higher fat intake (>4 weeks). Nevertheless, the long-term adaptation to a fat-rich diet is a crucial prerequisite for maintaining the capacity to perform endurance or high-intensity intermittent exercise (Helge, 2002; Volek et al., 2016). This is also apparent in our data from the 30-15_IFT_ where a considerable drop in TTE was found after 2 weeks on the VLCHF diet intervention (Figure 5); a response that was absent toward the end of the intervention period, suggesting normalization of glycogen stores due to enhanced gluconeogenesis (Veldhorst et al., 2009; Keller, 2011). The ability to maintain or even improve performance in the GXT or 30-15_IFT_ in the VLCHF group (Figure 7, 8) might be explained, first, by the fact that the endogenous CHO stores are not necessarily completely depleted when CHO intake is reduced (Volek et al., 2016), evidenced by the normal lactate response. Second, blood glucose concentration can also be maintained through glucose synthesis via a variety of substrates (Soeters et al., 2012). However, we prescribed a high-intensity but relatively short (up to 25 min) exercise bout that may not have been long enough to exhaust the glycogen stores, regardless of how much glycogen stores were diminished through CHO intake reduction. Therefore, it remains an open question whether this effect of VLCHF diet holds true for longer exercise (>25 min) bouts with high-intensity periods.

**FIGURE 9:**
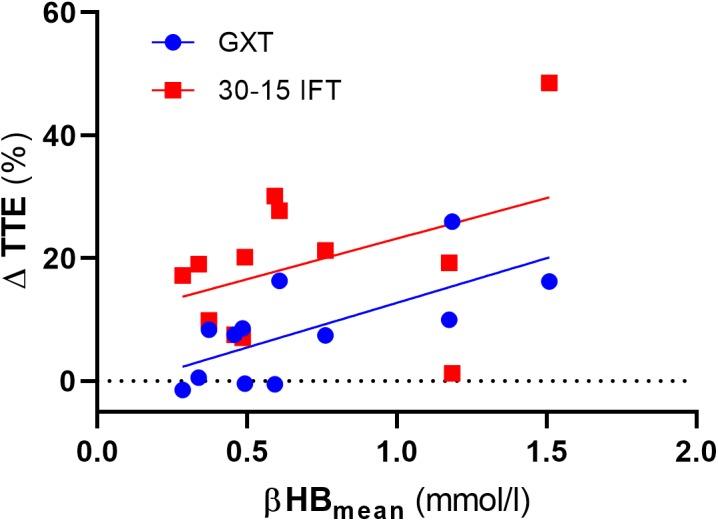
The association between total time to exhaustion (TTE) changes (%) and mean capillary β-hydroxybutyrate (βHB) concentration across the entire VLCHF diet intervention period. GXT, Graded Exercise Test; 30-15 IFT, 30-15 Intermittent Fitness Test.

We also analyzed the possible association between the level of capillary βHB concentrations and changes of TTE in both GXT and 30-15_IFT_. Due to substantial inter-individual diversity in capillary βHB concentrations, it might be expected that we can define responders vs. non-responders to the VLCHF diet within our investigated population. We found *moderate evidence* for a positive association between the mean level of βHB from the entire VLCHF diet intervention period and the PRE vs. 12 week change (%) in TTE_GXT_ [*r* = 0.69 (95% CL: 0.19, 0.91), *p* = 0.013, BF_10_ = 5.63]. However, there was only *anecdotal evidence* for this association with the performance change in 30-15_IFT_ (TTE_30-15IFT_; *p* = 0.185, BF_10_ = 0.78) (Figure 9). Ketone bodies (including βHB) are alternative energy substrates for brain and working muscles when the endogenous and exogenous CHO stores are reduced (Cox and Clarke, 2014). This might explain why participants with higher capillary βHB concentrations tended to be able to achieve the most substantial improvements in GXT performance. However, this explanation has limitations, since the capillary βHB concentration is a function of both βHB synthesis and utilization. Therefore, the association between βHB and its positive effect cannot be explained unequivocally by our data and warrants further research.

The obvious between-group difference was found in changes of relative $\dot{V}$O_2max_ (ml/kg/min). The $\dot{V}$O_2max_ *largely* increased in the VLCHF group despite a *trivial* change in the HD group. However, there was *moderate evidence* that $\dot{V}$O_2max_ was not changed in both groups when we expressed the marker in absolute values (l/min). Therefore, we ascribe this between-group difference in relative $\dot{V}$O_2max_ solely to the body mass reduction (Table 1) and not to potential maximal aerobic capacity enhancement in the VLCHF group. These findings correspond with our previous study, which showed similar $\dot{V}$O_2max_ changes in moderately trained individuals after a 4 week VLCHF diet intervention (Cipryan et al., 2018), and is in line with others (Zajac et al., 2014; Paoli et al., 2015), but contrary results have also been presented (Burke et al., 2017).

### RPE and HRV

Higher mental effort to sustain the scheduled exercise sessions, while consuming a short-term fat-rich diet compared with consuming a high CHO diet, was presented earlier by Helge (2002) for submaximal exercise until exhaustion. The significant between-group differences in the RPE data shown in this study did not occur until 45 min of the exercise (Helge, 2002). We showed that the RPE in both GXT and 30-15_IFT_ increased in both study groups but with no clear between-group differences (Table 3 for GXT, data for 30-15_IFT_ in Figure 5). Helge (2002) explained findings through the significantly higher catecholamine and heart rate response during submaximal exercise in subjects consuming a fat-rich diet. Those changes might be related to changes in ANS activity at rest and in response to exercise after a short-term reduction in CHO intake (increased sympathetic and possibly decreased parasympathetic response) (Pellizzer et al., 1999; Helge, 2002). The autonomic imbalance is a predictor of high blood pressure, hyperglycaemia, and a diagnosis of diabetes, cardiovascular disease and early mortality (Wulsin et al., 2016). As far as we know, we present novel data of the 12 week resting cardiac ANS activity in relation to the VLCHF diet. However, neither HR response to exercise (Figure 5) nor resting rMSSD (Figure 6) in our study showed any between-group differences. The vagal-related HRV parameter rMSSD remained stable throughout the experimental period, but a slight drop in the 2 week (also accompanied by performance reduction, see Figure 5) may support the necessity of an adequate adaptation to the VLCHF diet (Figure 6).

## Conclusion

A 12 week dietary CHO reduction with concomitant increases in fat intake in healthy young individuals did not impair high-intensity continuous or intermittent exercise lasting up to 25 min. The HRV assessment indicated, for the first time, that a 12 week exposure to the VLCHF diet combined with HIIT did not adversely affect ANS activity. Further, there was no evidence that individuals in the VLCHF group needed to raise their mental effort to achieve maximal performance. Finally, the similar PRE-POST average values of peak lactate indicate that the anaerobic energy contribution to performance was preserved in our adapted VLCHF group, and lowered RER values indicate enhancements in lipid metabolism throughout the exercise-intensity spectrum.

## Data Availability

The datasets generated for this study are available on request to the corresponding author.

## Ethics Statement

All participants provided written informed consent for the study protocol, which was approved by the local ethics committee and conformed to the principles outlined in the Declaration of Helsinki.

## Author Contributions

TD and LC designed the study, collected, analyzed, and interpreted the data, drafted, revised, and submitted the manuscript. DP, PH, and PL designed the study, interpreted the data, drafted, and revised the manuscript.

## Conflict of Interest Statement

The authors declare that the research was conducted in the absence of any commercial or financial relationships that could be construed as a potential conflict of interest.

## Footnotes

1 www.kaloricketabulky.cz
